# The Effects of St. John’s Wort and its Interactions with SSRI’s

**DOI:** 10.1192/j.eurpsy.2025.2087

**Published:** 2025-08-26

**Authors:** R. A. Maldonado-Puebla, N. Choudhury, B. Carr

**Affiliations:** 1 College of Osteopathic Medicine, Nova Southeastern University, Clearwater; 2 Psychiatry, University of Florida, Gainesville, United States

## Abstract

**Introduction:**

Hypericum perforatum, commonly known as St. John’s Wort, is a widely used herbal remedy for mild to moderate depression. It significantly affects drug metabolism by inducing the Cytochrome P450 enzyme system, particularly the CYP3A4 enzyme. This interaction can alter the metabolism of various medications, including oral contraceptives, cancer drugs, HIV antiretrovirals, and antidepressants. St. John’s Wort increases levels of serotonin, dopamine, and norepinephrine via reuptake inhibition, similar to the action of selective serotonin reuptake inhibitors (SSRIs). However, combining St. John’s Wort with SSRIs can dangerously elevate serotonin levels, potentially leading to serotonin syndrome, a serious and potentially life-threatening condition. This review explores the interactions between St. John’s Wort and SSRIs, focusing on metabolic effects and the risk of serotonin syndrome.

**Objectives:**

To determine the impact St. John’s Wort has on the metabolism of anti-depressants and to discover the differences in severity of serotonin syndrome between various SSRI’s.

**Methods:**

A comprehensive literature review was conducted using PubMed, Google Scholar, and Medline. The review focused on documented drug-drug interactions between St. John’s Wort and SSRIs, particularly their effects on the CYP enzyme system and the incidence of serotonin syndrome in patients taking both therapies.

**Results:**

The review identified that St. John’s Wort affects the metabolism of several antidepressants, primarily through the CYP3A4, CYP2C9, and CYP2C19 enzymes. SSRIs such as citalopram, escitalopram, and sertraline, metabolized by CYP2C19, are more likely to interact with St. John’s Wort than those metabolized by CYP2D6, such as paroxetine, fluoxetine, and fluvoxamine. The most significant adverse effect observed was serotonin syndrome, with case studies highlighting sertraline and paroxetine as the most commonly involved SSRIs. Dosages of SSRIs ranged from 20 mg to 75 mg, with St. John’s Wort dosages typically between 600 mg to 900 mg per day. All reported cases of serotonin syndrome involved both sertraline and paroxetine, suggesting that these SSRIs may have a higher risk when combined with St. John’s Wort, though a larger sample size is needed for statistical validation.

**Conclusions:**

The literature underscores the critical need to screen for patient’s who may have added St.John’s Wort into their treatment regimen, especially when taking SSRIs. St. John’s Wort can significantly alter the metabolism of SSRIs and increase the risk of serotonin syndrome. While interactions with sertraline and paroxetine are well-documented, further research is necessary to determine the risk profile of other SSRIs in combination with St. John’s Wort.

**Disclosure of Interest:**

None Declared

